# FGF21 and Cardiac Physiopathology

**DOI:** 10.3389/fendo.2015.00133

**Published:** 2015-08-31

**Authors:** Anna Planavila, Ibon Redondo-Angulo, Francesc Villarroya

**Affiliations:** ^1^Departament de Bioquímica i Biologia Molecular, Facultat de Biologia, Institut de Biomedicina de la Universitat de Barcelona, Universitat de Barcelona, Barcelona, Spain; ^2^CIBER Fisitopatologia de la Obesidad y Nutrición, Instituto de Salud Carlos III, Barcelona, Spain

**Keywords:** cardiac hypertrophy, sirtuins, PGC1alpha, cardiac pathology, oxidative stress

## Abstract

The heart is not traditionally considered either a target or a site of fibroblast growth factor-21 (FGF21) production. However, recent findings indicate that FGF21 can act as a cardiomyokine; that is, it is produced by cardiac cells at significant levels and acts in an autocrine manner on the heart itself. The heart is sensitive to the effects of FGF21, both systemic and locally generated, owing to the expression in cardiomyocytes of β-Klotho, the key co-receptor known to confer specific responsiveness to FGF21 action. FGF21 has been demonstrated to protect against cardiac hypertrophy, cardiac inflammation, and oxidative stress. FGF21 expression in the heart is induced in response to cardiac insults, such as experimental cardiac hypertrophy and myocardial infarction in rodents, as well as in failing human hearts. Intracellular mechanisms involving PPARα and Sirt1 mediate transcriptional regulation of the *FGF21* gene in response to exogenous stimuli. In humans, circulating FGF21 levels are elevated in coronary heart disease and atherosclerosis, and are associated with a higher risk of cardiovascular events in patients with type 2 diabetes. These findings provide new insights into the role of FGF21 in the heart and may offer potential therapeutic strategies for cardiac disease.

## Introduction

More than a decade has passed since fibroblast growth factor 21 (FGF21), the 21st member of the FGF family, was identified and cloned. Among FGFs, FGF21 has been shown to be a secreted protein that acts as a metabolic regulator and plays a role in the control of glucose homeostasis, insulin sensitivity, and ketogenesis (1, 2). FGF21 expression is under the control of peroxisome proliferator-activated receptor-α (PPARα), and the main site of its production and release into the blood is considered to be the liver (1, 3). Extra-hepatic tissues, such as white and brown adipose tissues and skeletal muscle, also express FGF21 (4–6). Endocrine actions of FGF21 include the promotion of glucose uptake by white adipocytes through induction of the glucose transporter, Glut1 (7), activation of brown fat thermogenic activity (8), and promotion of the appearance of brown fat-like cells in white fat – the so-called “browning” process (9). FGF21 also has autocrine/paracrine effects, such as induction of hepatic ketogenesis (1). The action of FGF21 on target cells requires FGF receptors (mainly FGFR1 and FGFR4 in adipose tissue and liver, respectively) and β-Klotho, a single-pass transmembrane protein that functions as an obligate cofactor for FGF21 signaling (10, 11). The heart was originally not considered an FGF21 target or source, primarily because of modest expression of mRNA for FGF21 and the transcript encoding β-Klotho, the obligate co-receptor for cellular responsiveness to FGF21 (12). Intriguingly, emerging studies have demonstrated that FGF21 is involved in regulating cardiac function. Here, we review recent advances that have led to the current awareness of the role of FGF21 in cardiac pathophysiology.

## Cardiac Effects of FGF21

**Figure 1:**
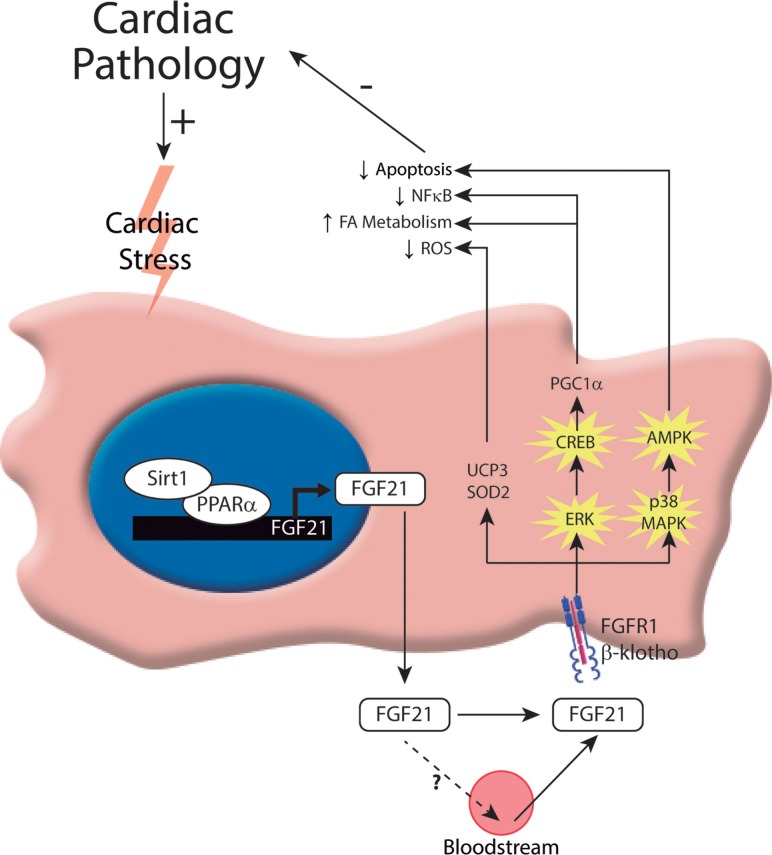
**Intracellular mechanisms involved in the control of FGF21 production and action on the heart**. In response to cardiac insults, cardiomyocytes induce the expression of FGF21. To date, one transcriptional pathway has been implicated in governing this process: the Sirt1–PPARα pathway. This pathway induces the expression of FGF21, which can be released by cardiac cells. Moreover, FGF21 acts on the heart, protecting it from cardiac damage. The molecular mechanisms involved in FGF21-mediated cardioprotection include activation of the FGF21 receptor, FGFR1, and the co-factor, β-Klotho, and subsequent activation of the ERK1/2 pathway. Phosphorylated CREB and p38-MAPK act through different intracellular mechanisms to exert protection against cardiac damage.

Despite initial evidence excluding the heart as a target tissue of FGF21, recent studies have demonstrated that FGF21 plays an important role in cardiac remodeling (13–16). A recent study from our laboratory provided the first report of the cardioprotective effects of FGF21 (13). This report showed that significant amounts of both the FGF21 receptor, FGFR1, and the co-factor, β-Klotho, are present at the protein level in cardiac cells. Moreover, treatment of cardiomyocytes in culture with FGF21 was found to activate the extracellular signal-regulated kinase (ERK) signaling pathway, which is considered the main intracellular pathway responsible for FGF21 intracellular actions. *In vivo*, hearts of FGF21-knockout mice exhibit an increase in relative weight and develop enhanced signs of dilatation. In response to isoproterenol infusion, a standard model used to induce cardiac hypertrophy, heart size, and cardiomyocyte volume are increased to a larger extent in FGF21-knockout mice compared with control mice. Furthermore, FGF21 treatment prevents cardiac hypertrophy development (at least, in neonatal mouse models), enhances fatty acid oxidation, and prevents the induction of pro-inflammatory pathways in the heart, thereby confirming the anti-hypertrophic properties of FGF21 (13). An exploration of the molecular mechanisms underlying these protective effects of FGF21 further revealed that isoproterenol treatment of FGF21-knockout mice upregulates pro-inflammatory markers in association with a decrease in PPARγ co-activator-1 α (PGC1α) expression levels. PGC1α is a transcriptional co-activator involved in the control of energy metabolism and oxidative stress in several tissues, including the heart (17). It was previously reported that cardiac expression of PGC1α is repressed by hypertrophic (18) and pro-inflammatory stimuli (19, 20). In this context, it was shown that the inhibitory action of FGF21 on cardiac hypertrophy and inflammation is associated with the induction of PGC1α (13). Moreover, FGF21 was shown to rapidly induce cAMP responsive element binding protein (CREB) phosphorylation in cardiomyocytes, a finding in accord with previous studies on FGFR1-mediated induction of CREB in other cell types (21). This latter effect may explain the observation that FGF21 induces the expression of PGC1α, a target of CREB (22) and an established repressor of the NF-κB pro-inflammatory pathway (23). Thus, this study indicates that FGF21 exerts protective effects against cardiac hypertrophy through a mechanism involving PGC1α (Figure 1).

Other studies have reported that FGF21 also exerts protection after myocardial infarction by inhibiting cardiomyocyte apoptosis (15), attenuating pathological myocardial remodeling, and reducing infarct size (16, 24). Furthermore, oxidative stress, which also plays a role in the pathogenesis of heart failure, is modulated by FGF21 (14). Cardiac FGF21 regulates genes involved in antioxidant pathways, thus preventing the production of reactive oxygen species (ROS) by cardiac cells. FGF21 induces the expression of genes encoding proteins involved in antioxidant pathways in cardiomyocytes in culture, especially uncoupling protein 3 (UCP3) and superoxide dismutase 2 (SOD2). Furthermore, the expression of antioxidant genes in response to signals that stimulate pro-oxidative/pro-inflammatory pathways in the heart is reduced in FGF21-knockout mice. Taken together, these very recent novel findings reveal new roles and mechanisms of action of FGF21 in the heart after myocardial infarction (Figure 1).

Finally, it has been proposed that FGF21 is involved in modulating cardiac lipid metabolism and homeostasis (25). In fact, the hormonal factor FGF21 is emerging as a key regulator of metabolism in general, providing significant health benefits and protective effects against metabolic disorders associated with obesity, such as insulin resistance, type II diabetes, and dyslipidemias. Recent initial pilot studies using an FGF21 analog to treat obese patients have shown promising results (26). Paradoxically, however, studies in both mice and humans have shown that obesity is associated with elevated circulating levels of FGF21, suggesting impaired FGF21 signaling (27–29). Thus, obesity has been proposed as an FGF21-resistance state. This phenomenon of reduced FGF21 action in obesity has been attributed to an abnormal reduction in the expression of the FGF21 co-receptor, β-Klotho, in white adipose tissue, commonly observed in rodent models of obesity and in obese individuals (30). In obese rats, β-Klotho protein expression in the heart is reduced, indicating that the FGF21-resistance state also occurs in the heart (24). Moreover, FGF21 deletion in mice exacerbates diabetic cardiomyopathy by aggravating cardiac lipid accumulation, although it is not clear whether this is a direct effect of the lack of FGF21 action on heart or indirect due to altered systemic lipid homeostasis in diabetic FGF21-null mice (31). A recent study has shown that FGF21 prevents diabetes-induced cardiac apoptosis by activating the ERK–p38MAPK–AMPK pathway (Figure 1); therefore, FGF21 has been proposed as a treatment for diabetes-related cardiac damage (32). Collectively, these data point to FGF21 as a key regulator of cardiac metabolism and a potential therapeutic target for the treatment of diabetic cardiomyopathy.

Other research approaches have evidenced mechanisms of myocardium protection by FGF21 (15). Myocardial ischemia activates innate protective processes not only in the heart but also in remote organs. Several recent investigations have demonstrated that the liver responds to ischemic myocardial injury by increasing the secretion of cardioprotective proteins (15, 33). One such protein identified by microarray-based gene expression profiling and protein analysis is FGF21 (33, 34), which was found to be highly increased at the protein level in the liver and also in adipose tissue after myocardial infarction. The authors of these latter studies also reported that this secreted FGF21 acts on ischemic cardiomyocytes to mitigate acute myocardial injury. Thus, systemic FGF21 generated mainly by the liver contributes to protection of the myocardium against ischemic damage.

In summary, current data support the idea that FGF21 acts directly on cardiac tissue to prevent the development of cardiac hypertrophy, reduce infarct damage, and attenuate the development of diabetic cardiomyopathy in animal models.

## Cardiac Production of FGF21

After the demonstration that FGF21 exerts cardioprotective effects, the next question raised was whether the heart was also able to endogenously produce FGF21. Our research group obtained the first data that addressed this question (13) showing that FGF21 is expressed in and secreted by cells of the heart in response to different cardiac stress stimuli, such as cardiac hypertrophy and myocardial infarction. FGF21 secreted by heart may function in an autocrine manner and, more unlikely, in an endocrine manner (see below). An analysis of different cell populations isolated from the heart showed that FGF21 is mainly produced by cardiomyocytes. Consistent with this, cardiomyocytes in culture were found to secrete FGF21 protein into the cell culture medium. Mouse models of experimentally induced cardiac hypertrophy and myocardial infarction also showed significant increases in FGF21 expression in the heart. Additional studies reported increases in cardiac FGF21 expression in obese rats (24), in type 1 diabetes (32), under fasting conditions (25), after endoplasmic reticulum (ER) stress (25), and in pro-oxidative/pro-inflammatory conditions (14). Interestingly, it has been shown that the human heart is also a source of FGF21, increasing in patients suffering from heart failure (14). Collectively, these studies indicate that FGF21 expression is induced in the heart in situations of pathological (e.g., infarct, hypertrophy) or physiological (e.g., fasting) stress, suggesting that FGF21 is a cardioprotective molecule secreted by the heart under conditions of stress.

Insight into the mechanism by which *FGF21* gene expression is regulated in the heart was provided by reports showing that the Sirt1–PPARα pathway is involved in the transcriptional control of *FGF21* (13, 14). This transcriptional pathway, which is also known to be involved in the regulation of FGF21 expression in the liver in the context of the control of carbohydrate and lipid metabolism (35), was found to play a pivotal role in controlling FGF21 expression and release in cardiac cells. Studies using genetic mouse models showed that cardiac FGF21 expression levels are reduced in PPARα-null mice and Sirt1-null mice compared with wild-type mice, confirming the involvement of this regulatory pathway in the control of cardiac *FGF21* gene expression. *In vitro* studies of cultured cardiomyocytes further showed that inhibition of PPARα clearly impairs the induction of FGF21 expression caused by Sirt1 overexpression, indicating that Sirt1 acts through PPARα (Figure 1).

Recently, cellular stresses, including ER stress and mitochondrial dysfunction, have been reported to induce FGF21 expression and release in several cellular systems, and there are indications that these same conditions induce FGF21 expression in the heart. In this context, ER stress inducers, such as the saturated fatty acid palmitate and the ER stressor tunicamycin, were shown to significantly increase FGF21 expression in cardiac cells in culture (26). Recent findings also underscore the potential role of FGF21 under conditions of mitochondrial dysfunction, with several studies showing that skeletal muscle is an FGF21-producing tissue in mice (36, 37) and humans (38), and that a mitochondrial respiratory chain deficiency leads to induction of FGF21 mRNA expression in skeletal muscle (39). In fact, pharmacological alteration of the mitochondrial respiratory chain in skeletal muscle cells in culture strongly induces FGF21 gene expression and FGF21 protein release into the cell culture medium (37). Moreover, it has been reported that muscle mitochondrial respiratory chain deficiencies in human patients are strongly correlated with increased plasma FGF21 levels (40). In addition, it has been recently reported that, in mouse cardiac tissue, mitochondrial dysfunction and stress responses lead to a dramatic – about 300-fold – induction of FGF21 (41).

The transcription factor, ATF4, might also be involved in the transcriptional control of cardiac *FGF21* expression in response to mitochondrial dysfunction or ER stress situations (25, 41). Most studies have proposed that binding of ATF4 to the *FGF21* gene promoter controls *FGF21* gene transcription in the heart and in skeletal muscle in response to signals elicited by mitochondrial dysfunction and ER stress. However, an ATF4-mediated pathway of *FGF21* induction by mitochondrial dysfunction involving increased ROS production has been found in skeletal muscle (36); whether this pathway operates in cardiac cells remains to be determined.

Although it is thought that the induction of FGF21 expression and release by skeletal muscle under conditions, such as pathogenic mitochondrial dysfunction, is responsible, in whole or in part, for the high systemic levels of FGF21 in patients with mitochondrial diseases, whether cardiac production of FGF21 results in altered systemic levels in physiological or pathogenic conditions studied to date remains unknown. The preponderance of currently available evidence clearly supports an autocrine role of FGF21 production by the heart.

## Cardiac FGF21: Autocrine Versus Endocrine Actions

The fact that the heart is both a target and a source of FGF21 raises the possibility of a potential autocrine loop for FGF21 in the myocardium. This possibility was first proposed in our recent study (13), where we showed that FGF21 is expressed in and secreted by cardiac cells in response to cardiac stress, and that secreted FGF21 was able to inhibit cardiac damage. This study demonstrated that the heart locally generates FGF21 via the Sirt1–PPARα pathway that acts in an autocrine manner to prevent hypertrophy, metabolic dysregulation, and activation of pro-inflammatory pathways in cardiac tissue. Collectively, the findings of this study describe a new mechanism for controlling cardiac inflammation and metabolism by locally produced FGF21. More recently, it was found that autocrine-acting FGF21 released by cardiomyocytes functions as an antioxidant factor in the heart, preventing ROS accumulation (14). In this autocrine loop, FGF21 is downstream of Sirt1, which is activated by upstream signals triggered by FGF21 released into the extracellular space (42). Thus, FGF21 release by cardiac cells appears to be both a cardiac response to oxidative stress and a signal to prevent ROS overflow. Future studies are expected to fill gaps in our current understanding of the relationship between oxidative stress and FGF21 expression.

In physiological models of cardiac hypertrophy, such as pregnancy (43), the circulating levels of FGF21 (44) as well as FGF21 expression levels in the heart are increased (45). In this physiological setting, both autocrine and endocrine cardioprotective roles of FGF21 might be at work. In contrast, in pathological hypertrophy, circulating levels of FGF21 are unchanged and FGF21 expression levels are increased only in cardiac tissue, indicating a predominant autocrine role of FGF21 in the heart in this pathological context (13).

The contribution of cardiac FGF21 production to the systemic pool remains to be elucidated. The magnitude of FGF21 expression by the heart compared with that of other tissues known to contribute to systemic FGF21, such as the liver, is relatively low (13), although a contribution of cardiac-derived FGF21 in pathological conditions, in which its production is increased, cannot be excluded. In this context, transgenic mice with cardiac-specific FGF21 overexpression show increased circulating FGF21 levels and altered body mass composition (26), suggesting that FGF21 released by the heart has the potential to act at a distance in an endocrine manner. However, more studies need to be done to elucidate possible endocrine actions of cardiac FGF21 and its actual contributions to whole-body homeostasis in physiological and naturally occurring pathological conditions.

**Figure 2:**
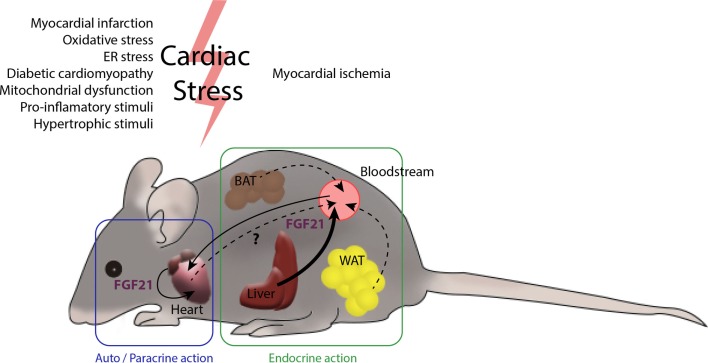
**The heart and FGF21 inter- and intra-organ communication**. Several tissues in the organism are potential producers of FGF21. The main contributor to circulating levels of FGF21 is the liver (46) and, under most physiological settings, a minor role is played by the white and brown adipose tissues. After myocardial infarction, liver and white adipose tissue produce large amounts of FGF21. The heart also produces increased levels of FGF21 after cardiac insults, such as cardiac hypertrophy, oxidative stress, and diabetes, among others. The endocrine action of FGF21 released by the liver and possibly by adipose tissues, together with autocrine FGF21 originating in heart itself, may act to protect against cardiac damage. The extent to which the heart contributes to systemic FGF21 levels is not yet fully established.

In summary, in addition to the protective role exerted by systemic, circulating FGF21 on the heart, local secretion of FGF21 in the context of cardiac damage may serve as an endogenous, autoregulatory, cardioprotective signaling pathway. Thus, systemic or locally generated FGF21 acts on the myocardium, protecting it from cardiac injury (Figure 2).

## FGF21 as a Biomarker for Cardiac Diseases

Several recent clinical studies in humans have explored the role of FGF21 in cardiovascular diseases. As previously mentioned, serum levels of FGF21 are elevated in subjects with adverse lipid profiles, obesity, metabolic syndrome, impaired glucose tolerance, type 2 diabetes mellitus, and hypertension (47, 48). Elevated serum FGF21 levels have also been recently reported in subjects with coronary heart disease or carotid artery plaques independently of established cardiovascular risk factors, suggesting its potential role as a biomarker for atherosclerotic diseases (49–51). Moreover, plasma FGF21 levels have been linked to a higher risk of cardiovascular events in patients with type 2 diabetes (52). In addition, serum FGF21 levels are elevated in atrial fibrillation patients in association with atrial remodeling (53). Interestingly, a recent study reported that serum FGF21 levels are independently associated with acute myocardial infarction (54), showing that serum FGF21 levels are markedly increased on the first day after the onset of myocardial infarction and remain high on days 3 and 7; moreover, FGF21 levels were found to be closely related to those of brain natriuretic protein (BNP), a common marker of cardiac diseases. The authors of this study concluded that high levels of FGF21 might be related to the incidence of re-infarction within 30 days after onset. Collectively, these studies suggest that FGF21 is a potential new biomarker for cardiac diseases.

## Conclusion and Perspectives

Several lines of evidence indicate that communication among cardiac cells via secreted factors may contribute to myocardial hypertrophic remodeling (55, 56). Recently, the term cardiomyokine has emerged to describe proteins secreted by the heart that have autocrine, paracrine, and/or endocrine functions crucial for the maintenance of cardiac function (57). Cardiomyokines have been estimated to number between 30 and 60, and include growth factors, endocrine hormones, and cytokines (58, 59). The findings summarized in this review establish FGF21 as a new cardiomyokine crucial for maintaining cardiac function. From a biomedical point of view, the possibility of preventing or even reversing pathological cardiac states and thereby slowing the development of heart disease is of utmost importance. Collectively, the findings summarized here indicate positive effects of FGF21 on the heart in the context of pathological conditions. Further research is warranted to explore FGF21 as a tool in the development of medical strategies to prevent and/or treat cardiac damage as well for use as a potential biomarker for cardiac diseases. In any case, the intracellular responses evoked by FGF21 represent a new rationale for developing a novel treatment modality based on potentiation of endogenous defenses in diseased hearts.

## Conflict of Interest Statement

The authors declare that the research was conducted in the absence of any commercial or financial relationships that could be construed as a potential conflict of interest.
